# Some Practical Notes

**Published:** 1909-08-28

**Authors:** 


### SOME PRACTICAL NOTES.

In replacing sparking plugs in hot cylinders they should not be screwed up too tightly or considerable difficulty will be experienced later in any attempt to remove them.

---

Novices will do well to remember that it is always safer to declutch when turning a corner, as the car can be more readily brought to a stand by the brakes, and, furthermore, the rear wheels can roll round the curve without slipping, while with the engine driving there is a decided tendency for the inner rear wheel to slip, which, of course, in time wears the tyres.

---

Trouble is often experienced with magnetos owing to over-lubrication. Sufficient oil to lubricate the bearings properly is all that is required, and any excess will sooner or later find its way on to the contact breaker points, and interfere to a greater or lesser extent with the contact. This in turn prevents the proper working of the machine.

---

It occasionally happens that the stem of an exhaust valve is somewhat longer than it should be, so that the valve is prevented from closing properly. The spare exhaust valve, which should invariably be carried, ought always to be examined for this defect (which is best done by placing the valve in position and seeing whether, when it is closed, there is the usual clearance between the end of the stem and the tappet), and if found too long it should be filed off.

A very handy thing for the motorist to keep in his tool-box is an assortment of wire nails of various sizes. These nails may be used for a variety of purposes, especially for replacing split pins, etc.

---

The cutting of motor tyres by the rims is generally caused either by overloading or lack of sufficient inflation. If the tyres are called upon to carry a greater load than their dimensions are calculated to bear no amount of inflation will keep them from flattening under the excessive load. This invariably results in the cracking and breaking down of the cover at its weakest point—where the flange engages the beaded edge. Rusty rims are also to be avoided, and they should occasionally be gone over and cleaned of any rust that may have accumulated. A coat or two of enamel will often prevent further corrosion, or the rims may be given a coat of wax. This is a satisfactory way of treating the rusty rims of an old car. The metal rims should be well scraped and sand-papered, and the wax should be heated and applied in a liquid state. It will not injure the rubber, and by keeping out the air it prevents further rusting of the metal. The surface of the rim which comes in contact with the inner tube should be smooth. If rough, it is likely to damage the tube, in which case the rim should be wrapped with a layer or two of tape, the loose ends being solutioned in place.

